# Decreased Electroencephalography Global Field Synchronization in Slow-Frequency Bands Characterizes Synaptic Dysfunction in Amnestic Subtypes of Mild Cognitive Impairment

**DOI:** 10.3389/fnagi.2022.755454

**Published:** 2022-04-08

**Authors:** Una Smailovic, Daniel Ferreira, Birgitta Ausén, Nicholas James Ashton, Thomas Koenig, Henrik Zetterberg, Kaj Blennow, Vesna Jelic

**Affiliations:** ^1^Division of Clinical Geriatrics, Center for Alzheimer Research, Department of Neurobiology, Care Sciences and Society, Karolinska Institutet, Huddinge, Sweden; ^2^Department of Clinical Neurophysiology, Karolinska University Hospital, Stockholm, Sweden; ^3^Department of Radiology, Mayo Clinic, Rochester, MN, United States; ^4^Clinic for Cognitive Disorders, Karolinska University Hospital-Huddinge, Stockholm, Sweden; ^5^Women’s Health and Allied Health Professionals Theme, Medical Unit Medical Psychology, Karolinska University Hospital, Huddinge, Sweden; ^6^Department of Psychiatry and Neurochemistry, Institute of Neuroscience and Physiology, The Sahlgrenska Academy at the University of Gothenburg, Mölndal, Sweden; ^7^Clinical Neurochemistry Laboratory, Sahlgrenska University Hospital, Mölndal, Sweden; ^8^Wallenberg Centre for Molecular and Translational Medicine, University of Gothenburg, Gothenburg, Sweden; ^9^King’s College London, Institute of Psychiatry, Psychology and Neuroscience, Maurice Wohl Institute Clinical Neuroscience Institute, London, United Kingdom; ^10^NIHR Biomedical Research Centre for Mental Health and Biomedical Research Unit for Dementia at South London and Maudsley NHS Foundation, London, United Kingdom; ^11^Psychiatric Electrophysiology Unit, Translational Research Center, University Hospital of Psychiatry, Bern, Switzerland; ^12^Department of Neurodegenerative Disease, UCL Queen Square Institute of Neurology, Queen Square, London, United Kingdom; ^13^UK Dementia Research Institute at UCL, London, United Kingdom; ^14^Hong Kong Center for Neurodegenerative Diseases, Hong Kong, Hong Kong SAR, China

**Keywords:** electroencephalography, synaptic dysfunction, amnestic mild cognitive impairment (aMCI), Alzheimer’s disease, EEG power, global field synchronization (GFS)

## Abstract

**Background:**

Mild cognitive impairment (MCI) is highly prevalent in a memory clinic setting and is heterogeneous regarding its clinical presentation, underlying pathophysiology, and prognosis. The most prevalent subtypes are single-domain amnestic MCI (sd-aMCI), considered to be a prodromal phase of Alzheimer’s disease (AD), and multidomain amnestic MCI (md-aMCI), which is associated with multiple etiologies. Since synaptic loss and dysfunction are the closest pathoanatomical correlates of AD-related cognitive impairment, we aimed to characterize it in patients with sd-aMCI and md-aMCI by means of resting-state electroencephalography (EEG) global field power (GFP), global field synchronization (GFS), and novel cerebrospinal fluid (CSF) synaptic biomarkers.

**Methods:**

We included 52 patients with sd-aMCI (66.9 ± 7.3 years, 52% women) and 30 with md-aMCI (63.1 ± 7.1 years, 53% women). All patients underwent a detailed clinical assessment, resting-state EEG recordings and quantitative analysis (GFP and GFS in delta, theta, alpha, and beta bands), and analysis of CSF biomarkers of synaptic dysfunction, neurodegeneration, and AD-related pathology. Cognitive subtyping was based on a comprehensive neuropsychological examination. The Mini-Mental State Examination (MMSE) was used as an estimation of global cognitive performance. EEG and CSF biomarkers were included in a multivariate model together with MMSE and demographic variables, to investigate differences between sd-aMCI and md-aMCI.

**Results:**

Patients with sd-aMCI had higher CSF phosphorylated tau, total tau and neurogranin levels, and lower values in GFS delta and theta. No differences were observed in GFP. The multivariate model showed that the most important synaptic measures for group separation were GFS theta, followed by GFS delta, GFP theta, CSF neurogranin, and GFP beta.

**Conclusion:**

Patients with sd-aMCI when compared with those with md-aMCI have a neurophysiological and biochemical profile of synaptic damage, neurodegeneration, and amyloid pathology closer to that described in patients with AD. The most prominent signature in sd-aMCI was a decreased global synchronization in slow-frequency bands indicating that functional connectivity in slow frequencies is more specifically related to early effects of AD-specific molecular pathology.

## Introduction

Mild cognitive impairment (MCI) is an intermediate stage between cognitively healthy brain aging and dementia (Winblad et al., 2004) and is one of the most common diagnoses in memory clinic (Wahlund et al., 2003). It represents a risk condition for future development of dementia, with an annual conversion rate ranging from 5 to 15% (Bruscoli and Lovestone, 2004; Farias et al., 2009). The risk of progression to dementia is even higher among patients with MCI from the specialized memory clinics than community-based populations, emphasizing the need for improved clinical phenotyping of patients with objectively evident cognitive impairment (Mitchell and Shiri-Feshki, 2009). Several diagnostic criteria for MCI have been proposed so far (Petersen, 2004; Winblad et al., 2004; Albert et al., 2011), all of which highlight the heterogeneity of this condition in terms of its clinical and etiological presentation. MCI is typically classified as amnestic or non-amnestic, depending on whether there is an objectively evident impairment in the memory domain (Petersen, 2004, 2016; Winblad et al., 2004). Amnestic MCI (aMCI) is considered to clinically correspond to the prodromal stage of typical Alzheimer’s disease (AD) (Dubois et al., 2014; Petersen, 2016) and has been linked to the AD biomarker profile including positive markers for amyloid and tau pathology (Visser et al., 2009; Wolk et al., 2009). MCI can be additionally classified as a single or multiple domain based on the number of affected cognitive domains, with the latter including deficits in memory, language, attention, executive function, and visuospatial skills (Petersen, 2004; Albert et al., 2011). Objectively verified impairment in multiple cognitive domains seems to be related to the faster progression to dementia, including dementia due to AD, Lewy bodies (DLB), and cerebrovascular disease (Petersen, 2004; Hughes et al., 2011).

Cognitive subtypes of MCI still exhibit variability in terms of disease etiology and prognosis, emphasizing the role of biomarkers in delineating more homogeneous subgroups of patients with objective cognitive impairment. Recent studies have shown that markers of synaptic degeneration and dysfunction are closely related to cognitive impairment (Scheff et al., 2006, 2007; Headley et al., 2018) and future cognitive deterioration in patients with MCI (Poil et al., 2013; Kvartsberg et al., 2015a), supporting their role in characterizing subgroups of patients with cognitive impairment.

Electroencephalography (EEG) is a neurophysiological method that can detect real-time changes in the brain synaptic activity associated with different vigilance states, cognitive load, and pathological brain disorders. Its clinical use spans across a spectrum of brain disorders with underlying synaptic pathology that causes cortical hypo- and hyperexcitability, focal, or more generalized cerebral dysfunction (Schomer and Lopes da Silva, 2015). The nature of cortical and subcortical synaptic degeneration and loss in patients with cognitive impairment therefore suggest EEG as a candidate neurophysiological marker of impaired cerebral activity. So far, most of the research studies have emphasized the advantage of quantitative EEG (qEEG) that offers objective, comprehensive, and more generalizable interpretation of EEG analyses (Smailovic and Jelic, 2019). The quantitative resting-state EEG analysis commonly assesses the power and synchronization of EEG oscillations across four conventional frequency bands that are also routinely described during visual EEG assessments (Schomer and Lopes da Silva, 2015). The most common qEEG finding in patients with cognitive impairment includes the increase in power in slow-frequency bands (i.e., delta and theta) and decrease in power in fast-frequency bands (i.e., alpha and beta) (Smailovic and Jelic, 2019). At the same time, the decrease in global EEG synchronization has also been reported in patients with cognitive impairment, noted as early as in patients with subjective cognitive decline (SCD) (Koenig et al., 2005). In the context of MCI subtypes, different qEEG changes have been reported in relation to the underlying neurodegenerative or cerebrovascular pathology (Moretti et al., 2012; Schumacher et al., 2020) and duration of disease symptoms (Moretti et al., 2010).

Synaptic dysfunction in patients with cognitive impairment can be further assessed by changes in molecular markers available from cerebrospinal fluid (CSF) that are thought to reflect degeneration and loss of pre- or postsynaptic compartments in the central nervous system. Recent studies support neurogranin, a postsynaptic neuron-specific protein, as a CSF marker of synaptic degeneration in AD (Kvartsberg et al., 2015b; Portelius et al., 2018). Neurogranin is mainly expressed in the cortical and hippocampal neurons and has an important role in regulating synaptic plasticity (Bogdanovic et al., 2002; Zhong et al., 2009; Zhong and Gerges, 2012). Previous studies have shown that increased neurogranin levels in the CSF correlate with poor memory scores and aMCI presentation (Lista et al., 2016; Headley et al., 2018) as well as progression to AD dementia in patients with MCI (Kvartsberg et al., 2015a).

Despite the close relationship between synaptic markers and measures of cognitive impairment, their role in characterizing heterogeneous clinical presentation of the mild neurocognitive disorders is yet to be fully elucidated. The main aim of this study was to investigate whether EEG power and synchronization and novel CSF synaptic marker neurogranin, in addition to the conventional CSF markers of amyloid and tau pathology, differentiate subtypes of aMCI based on the single- vs. multidomain cognitive profile. We hypothesized that neurophysiological and molecular markers of synaptic dysfunction have added value to conventional AD biomarkers in characterizing aMCI subtypes.

## Materials and Methods

### Study Population

The study included 82 patients from memory clinic recruited at Karolinska University Hospital and diagnosed with MCI based on the clinical criteria by Winblad et al. (2004). Our comprehensive clinical assessment included clinical interviews with the patient and informant, blood testing, lumbar puncture, screening for depression, and somatic and neurological examinations. MCI of the amnestic type has been defined during a discussion on the consensus diagnostic round and was based on the clinical observation and summarized neuropsychological test profile. Patients with an amnestic profile of MCI were further clinically subtyped into a single domain (sd-aMCI; *n* = 52) and multiple-domain amnestic MCI (md-aMCI: *n* = 30) based on the standard neuropsychological examination including tests of language, visuospatial ability, executive functions, and memory (Table 1; Ekman et al., 2020). Impairment in memory and/or any other cognitive domain was standardized by *z*-transformation of test results, using age- and education-adjusted Swedish norms and references (Arnáiz and Almkvist, 2003; Wechsler, 2003, 2010). Clinical Dementia Rating (CDR) scale was used to assess the level of disease severity. The CDR global score was 0 or 0.5 with no major difficulties in performing independent activities of daily living. Global cognitive performance was estimated by means of the Mini-Mental State Examination (MMSE) (Folstein et al., 1975).

**TABLE 1:** Cognitive tests used for subtyping of MCI patients into sd-aMCI and md-aMCI groups in the current study.

Cognitive domains	Neuropsychological tests
Language	WAIS-IV: Similarities; BNT; Letter Fluency (F-A-S); Semantic Fluency (animals)
Visuospatial	WAIS-IV: Block Design; RCFT; Copying Geometric Shapes; Clock Drawing/Reading Test
Executive	WAIS-IV: Digit Symbol; Trail-Making Test A&B; D-KEFS: Trail-Making Test 1–5
Attention/Working memory	WAIS-IV: Digit span and Arithmetic; RCFT; WMS-III: Logical Memory
Semantic/Episodic memory	WAIS-IV: Information; RAVLT; RCFT; WMS-III: Logical Memory

*BNT, Boston Naming Test; D-KEFS, Delis-Kaplan Executive System; MCI, mild cognitive impairment; RCFT, Rey-Osterrieth Complex Figure Test; WAIS-IV, Wechsler Adult Intelligence Scale 4th edition; WMS-III, Wechsler Memory Scale 3rd edition; md-aMCI, multidomain amnestic MCI; sd-aMCI, single-domain amnestic MCI.*

All patients underwent lumbar puncture and CSF conventional (i.e., Aβ42, p-tau, and t-tau) and synaptic (i.e., neurogranin) biomarker analysis and resting-state EEG recording at the baseline. The exclusion criteria involved patients younger than 50 years, presence of any major psychiatric or neurological disorder, brain trauma, psychotropic medication, and the time gap between the EEG recording and lumbar puncture longer than 6 months. Demographics and clinical data in the whole MCI cohort as well as in sd-aMCI and md-aMCI subgroups are presented in Table 2. We also presented descriptive data for a selection of neuropsychological tests within different cognitive domains to illustrate the differences between the sd-aMCI and md-aMCI groups (Table 3). The study was approved by the local ethical committee of the Karolinska Hospital and Regional Ethical Review Board in Stockholm (Dnr: 2020-00678, 2011/1978-31/4).

**TABLE 2:** Demographics and clinical characteristics in the whole MCI cohort and sd-aMCI and md-aMCI subgroups.

Variables	Whole cohort (*N* = 82)	sd-aMCI (*n* = 52)	md-aMCI (*n* = 30)	Effect size (η^2^)	*p*-value
Age, years	65.49 (7.42)	66.85 (7.31)	63.13 (7.12)	0.059	**0.028**
Sex, women (%)	52%	52%	53%	0.001	0.999
Education, years	12.58 (3.94)	12.18 (3.31)	13.27 (4.83)	0.018	0.281
MMSE	27.31 (1.94)	27.65 (1.67)	26.73 (2.24)	0.053	**0.040**

*Data presented as mean and standard deviation except for sex, where percentage of women is presented. p-values were obtained using t-tests (or ANCOVA when including age as a covariate) for all the variables except for sex, where the chi-square test was used. MMSE, Mini-Mental State Examination. sd-aMCI, single-domain amnestic MCI; md-aMCI, multidomain amnestic MCI.*

**TABLE 3:** Neuropsychological test results in *z*-scores for subtyping of MCI patients into sd-aMCI and md-aMCI groups.

Cognitive domains/ Neuropsychological tests	sd-aMCI (*n* = 52)	md-aMCI (*n* = 30)
**Language**		
Similarities	0.23 (0.89)	−0.28 (0.79)
**Visuospatial**		
W: Block Design	0.24 (1.05)	−0.63 (0.80)
RCFT, copy	−0.69 (0.97)	−1.83 (2.14)
**Executive**		
W: Digit symbol	−0.52 (0.89)	−1.02 (0.87)
**Attention/Working memory**		
Digit span	−0,32 (0.85)	−0.94 (0.64)
**Episodic memory**		
RAVLT, total learning	−1.37 (0.84)	−1.32 (0.96)
RAVLT, delayed recall	−1.71 (0.86)	−1.87 (0.81)
RCFT, immediate recall	−0.88 (1.16)	−1.24 (1.03)

*Data presented as mean and standard deviation. RAVLT, Rey Auditory Verbal Learning Test; W, Wechsler Adult Intelligence Scale (WAIS) 3rd and 4th edition.*

### Cerebrospinal Fluid Sampling and Analysis

All CSF samples were collected according to the standard lumbar puncture procedure (Engelborghs et al., 2017). Conventional markers of AD (i.e., Aβ42, t-tau, and p-tau) were analyzed using the xMAP technology and INNO-BIA AlzBio3 kit (Innogenetics) (Olsson et al., 2005). The clinical cutoff value for amyloid positivity according to the CSF Aβ42 levels was < 550 ng/L. Neurogranin concentrations in the CSF were analyzed using the in-house-developed ELISA assay as described previously in detail by Kvartsberg et al. (2019).

### Electroencephalography Recordings and Analysis

All MCI patients underwent resting-state EEG recording within 6 months of lumbar puncture and CSF sampling. Resting-state EEGs were recorded as a standard clinical procedure for 15–20 min on the nervous system at the Department of Clinical Neurophysiology at Karolinska University Hospital (NicoletOne EEG Reader v5.93.0.424, Natus NicoletOne, Pleasanton, CA) using the standard placement of 21 scalp electrodes according to the 10/20 system. Trained biomedical engineers were noting any changes in the vigilance states and alarming patients in the case of drowsiness during EEG recording. The standard recording setup was described previously in detail by Smailovic et al. (2018).

All EEGs were exported in the common average reference montage and preprocessed following the same procedure. All exceptional events during the resting-state eyes-closed recording, such as periods of eyes opening, drowsiness, alarming of the patient, movements, and other non-physiological and physiological artifacts, were removed by visual inspection and manual artifact rejection. Ocular artifacts were additionally removed by using electrooculogram (EOG) and semi-automated independent component algorithm (ICA). Preprocessed EEGs were analyzed in frequency-transformed artifact-free 2 s EEG epochs and averaged within subjects. The qEEG analysis involved two complementary and comprehensive EEG measures of global field power (GFP) and global field synchronization (GFS). GFP reduces and summarizes data across multiple EEG channels to a single measure of generalized EEG amplitude. Specifically, in the context of this study, GFP corresponds to the root mean of spectral amplitudes across all EEG channels (Huang et al., 2000; Michel, 2009). GFS, in contrast, reflects, for a particular frequency, the amount of the EEG activity that can be explained by a common phase across all EEG electrodes (Koenig et al., 2001). The computation of GFS measure has been introduced and described in detail in Koenig et al. (2001). GFP and GFS measures were averaged in predefined conventional frequency bands defined within the frequencies as follows: delta (1–3.5 Hz), theta (4–7.5 Hz), alpha (8–11.5 Hz), and beta (12–19.5 Hz). The beta frequency range was defined between 12 and 20 Hz since EEG frequencies above 20 Hz may be contaminated with muscle artifacts (Goncharova et al., 2003; Whitham et al., 2007).

### Statistical Analysis

We compared sd-aMCI and md-aMCI groups with *t*-tests when the dependent variables were continuous and chi-square tests when the dependent variables were categorical. We applied the Mann-Whitney *U*-test for group differences when continuous variables were not normally distributed. We also used analysis of variance (ANCOVA) to compare sd-aMCI and md-aMCI groups in MMSE scores while controlling for the effect of age as a covariate. Effect sizes are reported as eta squared (η^2^) and interpreted per convention: small = 0.01, medium = 0.06, and large = 0.14. We further wanted to compare EEG measures with CSF biomarkers and key clinical measures, such as MMSE, in their capacity to differentiate sd-aMCI from md-aMCI. For this analysis, we used MMSE instead of the comprehensive neuropsychological protocol to avoid circularity, since the MCI subtype was based on the neuropsychological protocol. Age, sex, and education were also included to assess their role in the model. Given the nature of our variables, the multicollinearity between several of the variables, and the sample size, we chose to apply a classification random forest model, which is superior to the general linear model and other statistical methods in such a scenario (Breiman, 2001; Machado et al., 2018). Random forest is an ensemble method in machine learning based on growing of multiple decision trees *via* bootstrap aggregation (i.e., bagging). Each tree predicts a classification independently and votes for the corresponding class. The best model for each outcome variable is chosen from the majority of votes. The combination of bootstrap aggregation (Breiman, 1996) with random feature selection (Amit and Geman, 1997) in a random forest is important to prevent data overfitting and increase the prediction power. Our random forest model included 5,000 trees, providing an accurate estimation of the importance of variables without introducing too much noise in the model due to the addition of redundant trees. Each of the trees was trained on randomly selected 70% of the data and subsequently tested on the unseen 30% of the data. A total of three variables were randomly selected and tested at each split, where the number of variables was defined by the square root of the total number of predictors in the model. The maximum depth of each tree was determined by the maximum number of nodes in each tree, ensuring at least one observation per node (i.e., trees were not truncated at a given depth). We conducted a random forest classification model (Liaw and Wiener, 2002), with the MCI subtype (i.e., sd-aMCI vs. md-aMCI) treated as the outcome variable, and age, sex, education, MMSE, CSF amyloid-beta 42, CSF p-tau, CSF t-tau, CSF neurogranin, and the four GFP and four GFS qEEG measures included as the predictors. We accounted for the fact that the outcome variable presented with an unbalanced number of cases in its two levels (i.e., sd-aMCI *n* = 52 and md-aMCI *n* = 30). When the groups are not balanced in size, the probability of taking observations from the larger group is higher, which could affect the performance of the model. Therefore, we *a priori* fixed our model to select random samples of the two MCI groups that were 50/50 in proportion. We reported the classification error as a measure of goodness of the model (i.e., out-of-the-bag estimated error rate, OOB-EER) (Breiman, 2001). When outcome variables are dichotomous, as it is our case, the error by chance is 50%. Therefore, a classification error below 50% is better than chance, with values closest to 0% denoting better classification performance, hence, good reliability of the model. We also reported the importance of the predictors as a measure of their contribution toward differentiating the sd-aMCI and md-aMCI groups. Higher important values denote a stronger contribution to the prediction. The random forest model was further complemented with the Pearson correlation coefficients (or point biserial correlation in the case of categorical variables, which were coded as dummy variables), to present the magnitude and direction of the association between variables (i.e., bivariate association). All the analyses were performed using the R^1^ version 3.2.4 software, with a *p*-value ≤ 0.05 deemed significant.

## Results

### Demographics and Clinical Characteristics

With respect to the demographical characteristics, patients in the md-aMCI group were significantly younger (63.1 ± 7.1 years) than patients in the sd-aMCI group (66.9 ± 7.3 years). There were no statistically significant differences in the distribution of sex and years of education between the two groups. However, patients in the md-aMCI group obtained significantly lower MMSE scores (26.7 ± 2.2) than patients in the sd-aMCI group (27.7 ± 1.7) (*p* = 0.040) (Table 2). ANCOVA showed that group differences in MMSE scores remained significant when including age as a covariate (*p* = 0.040). The differences in age and MMSE were, however, small, with effect sizes (η^2^) below 0.06. Results of the neuropsychological test presented in *z*-scores for patients with sd-aMCI and md-aMCI are presented in Table 3.

### Conventional and Synaptic Cerebrospinal Fluid Biomarkers

The analysis of conventional AD CSF biomarkers revealed that patients from the sd-aMCI group had higher CSF t-tau (*p* = 0.009) and p-tau levels (*p* = 0.031) than patients from the md-aMCI group. Even though the sd-aMCI group exhibited lower CSF Aβ42 levels and included a higher percentage of patients with CSF amyloid positive than those in the md-aMCI group, the difference was not statistically significant in the patient cohort of this study. In contrast, neurogranin levels were significantly increased in the CSF of patients with sd-aMCI compared with those with md-aMCI (*p* = 0.044) (Table 4).

**TABLE 4:** Conventional and synaptic CSF biomarkers in the whole MCI cohort and sd-aMCI and md-aMCI subgroups.

Variables	Whole cohort (*N* = 82)	sd-aMCI (*n* = 52)	md-aMCI (*n* = 30)	Effect size (η^2^)	*p*-value
CSF amyloid-beta 42 (ng/L)	697 (255.6)	673 (249.7)	738 (264.8)	0.015	0.273
CSF amyloid-beta 42, abnormal (% positive)	33%	35%	30%	0.002	0.854
CSF t-tau (ng/L)	397 (212.7)	443 (231.9)	317 (145.9)	0.082	**0.009**
CSF p-tau (ng/L)	66 (25.3)	70 (26.5)	58 (21.4)	0.057	**0.031**
CSF neurogranin (ng/L)	204 (75.1)	217 (80.3)	182 (60.2)	0.050	**0.044**

*Data presented as mean and standard deviation except for CSF amyloid-beta 42, abnormal where percentage of a positive biomarker is presented. p-values were obtained using t-tests for all the variables except for CSF amyloid-beta 42, abnormal, where the chi-square test was used. The cutoff value for CSF Aβ42 positivity < 550 ng/L. CSF, cerebrospinal fluid. sd-aMCI, single-domain amnestic MCI; md-aMCI, multidomain amnestic MCI.*

### Quantitative Electroencephalography Parameters in Single-Domain Amnestic Mild Cognitive Impairment and Multidomain Amnestic Mild Cognitive Impairment

The qEEG analysis showed that the sd-aMCI group had a statistically significant lower GFS delta (*p* = 0.029) and theta (0.023) compared with that of the md-aMCI group. There were no statistically significant differences in the EEG measure of global power (i.e., GFP) between the two groups, in any of the conventional frequency bands (Table 5).

**TABLE 5:** qEEG measures of global field power (GFP) and synchronization (GFS) in four conventional frequency bands in the whole MCI cohort and sd-aMCI and md-aMCI subgroups.

Variables	Whole cohort (*N* = 82)	sd-aMCI (*n* = 52)	md-aMCI (*n* = 30)	Effect size (η^2^)	*p*-value
GFP delta	0.102 (0.051)	0.098 (0.048)	0.109 (0.056)	0.010	0.367
GFP theta	0.055 (0.044)	0.054 (0.044)	0.058 (0.046)	0.002	0.717
GFP alpha	0.157 (0.120)	0.164 (0.135)	0.144 (0.090)	0.007	0.468
GFP beta	0.036 (0.029)	0.039 (0.032)	0.030 (0.023)	0.025	0.180
GFS delta	0.550 (0.026)	0.545 (0.022)	0.558 (0.030)	0.059	**0.029**
GFS theta	0.554 (0.026)	0.549 (0.024)	0.563 (0.027)	0.063	**0.023**
GFS alpha	0.576 (0.036)	0.575 (0.032)	0.578 (0.043)	0.002	0.719
GFS beta	0.516 (0.022)	0.516 (0.022)	0.518 (0.022)	0.002	0.720

*Data presented as mean and standard deviation. p-values were obtained using t-tests for all the variables.*

*GFP, global field power; GFS, global field synchronization. sd-aMCI, single-domain amnestic MCI; md-aMCI, multidomain amnestic MCI.*

### Classification Model for Differentiating Single-Domain Amnestic Mild Cognitive Impairment and Multidomain Amnestic Mild Cognitive Impairment

**FIGURE 1:**
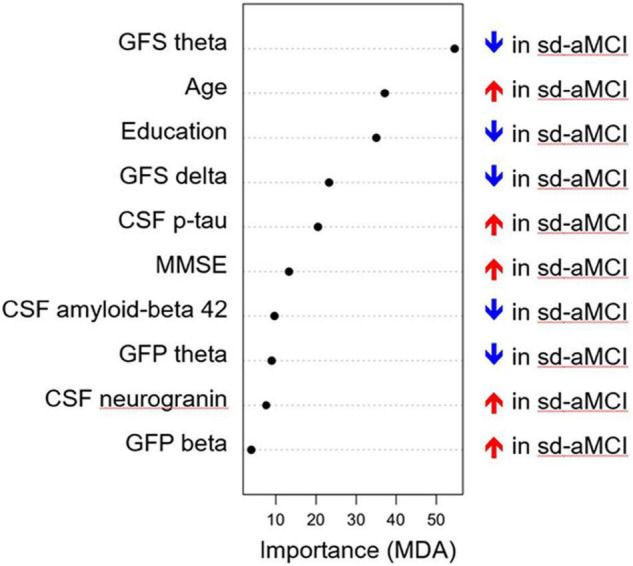
Classification model for differentiating sd-aMCI and md-aMCI. The *x*-axis displays the importance of the variables in the differentiation between sd-aMCI and md-aMCI, with higher values (i.e., dots to the right side) indicating a greater importance. MDA, mean decrease accuracy; GFP, global field power; GFS, global field synchronization; MMSE, Mini-Mental State Examination; MCI, mild cognitive impairment; md-aMCI, multidomain amnestic MCI; sd-aMCI, single domain amnestic MCI.

**FIGURE 2:**
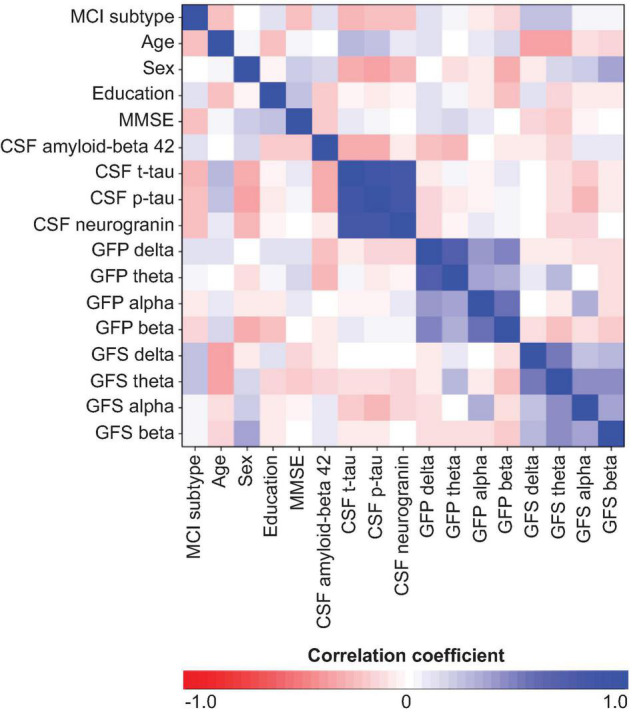
Correlation matrix between all predictors and outcome variable of the random forest model. MCI subtype was coded as sd-aMCI = 0 and md-aMCI = 1. Sex was coded as women = 0 and men = 1. GFP, global field power; GFS, global field synchronization; MMSE, Mini-Mental State Examination; CSF, cerebrospinal fluid.

The multivariate model showed a good performance (out-of-the-bag error = 35.5%). Figure 1 shows that several EEG measures had an important contribution toward discriminating the sd-aMCI and md-aMCI groups, including GFS theta and delta and GFP theta and beta. This contribution was beyond the differences in age and MMSE between the two MCI groups. Other measures that were important to differentiate sd-aMCI from md-aMCI were age, education, CSF p-tau, MMSE, CSF Aβ42, and neurogranin. Regarding the direction of these measures, lower GFS theta and delta, lower GFP theta, higher GFP beta, lower education, older age, higher MMSE scores, higher CSF p-tau and neurogranin, and lower CSF amyloid-beta 42 were almost always related to sd-aMCI (17.0% of classification error), while the opposite was not always true for md-aMCI (65.5% of classification error). Figure 2 shows the correlation matrix between all predictors and the outcome variable in our random forest model.

## Discussion

This study reports that the qEEG measure of global synchrony (i.e., GFS) in slow frequencies, in particular in the theta band, is the strongest discriminator between the two most common clinical subtypes of aMCI: single-domain (sd-aMCI) and multi-domain amnestic MCI (md-aMCI). The GFS in theta-frequency band was significantly lower in the sd-aMCI group than that of the md-aMCI group, followed by lower GFS in the delta band. These differences and the capacity of qEEG measures to discriminate between MCI groups were above and beyond group differences in MMSE and age. Patients with single-domain aMCI in this study, in accordance with the literature and the common clinical experience, had more pathological changes in CSF biomarkers of amyloid pathology and neurodegeneration (Visser et al., 2009; Damian et al., 2013). Interestingly, a previous study by Koenig et al. (2005) on GFS alterations on the clinical continuum of AD showed that the decrease in the alpha-frequency band was more pronounced than in other frequency bands, with a gradient mode of decrease across the severity of the functional decline. This might not be at odds with our findings in this study since patients with early AD and dementia show a shift of alpha power peak toward lower frequencies in the theta range (Samson-Dollfus et al., 1997; Moretti et al., 2004). It would have been of interest to subdivide alpha frequencies in slow and fast alpha bands since they could have different functional significance as suggested previously (Schomer and Lopes da Silva, 2015).

In the study by Koenig et al. (2005), 2-center large data sets from cognitively healthy subjects and patients ranging from subjective and MCI to the most severe stages of AD dementia were included although not with a balanced number of cases in different diagnostic categories. Thus, there was a noteworthy heterogeneity of the contrast groups phenotyped only by the clinical assessment and not by additional consideration of disease biomarkers. Furthermore, the inclusion of a considerable number of healthy subjects might have introduced a bias toward alpha frequencies, since the GFS in the alpha band was strongest in the healthy controls thus increasing contrast toward other groups.

Another EEG study that explored changes in global EEG synchronization in AD showed a decrease in GFS in beta-frequency band in patients with AD with more severe disease stages when compared with those in healthy controls (Ma et al., 2014). However, only GFS in the slow delta band correlated significantly with a CDR scale, a measure of clinical disease severity, which was also found in an earlier study by Park et al. (2008). Increased synchronization within and between frontal and parietal areas in the delta band has been further associated with better visual episodic memory performance (Tóth et al., 2012), while the overall increase in the EEG delta activity was observed during the performance of arithmetic tasks (Dimitriadis et al., 2010). Delta synchronization has been additionally related to the object maintenance in short-term memory in experiments involving primates (Siegel et al., 2009). These findings highlight the functional importance of slow-frequency synchronization for maintaining healthy cognitive performance.

Our findings are further supported by a plausible conceptual background. Amnestic syndrome in AD is driven by hippocampal dysfunction (Dubois et al., 2014), and it was shown that the source of theta activity originates in the hippocampus and entorhinal cortex (Schomer and Lopes da Silva, 2015). It is plausible that cortico-cortical disconnection in the limbic system is an early event in the pathophysiology of the amnestic syndrome. Although intraoperative recordings, as well as magnetoencephalography (MEG) studies, have confirmed the existence of hippocampal theta activity in human subjects (de Araújo et al., 2002; Jacobs and Kahana, 2010), it is still speculative to conclude that theta activity in our patient population has an exclusive hippocampal origin without in-parallel application of source imaging. Studies performed in rodents have shown that theta oscillations seem to coordinate the activity of widespread neural networks, such as prefrontal, somatosensory, and entorhinal cortices (Chrobak and Buzsáki, 1998; Siapas et al., 2005; Sirota et al., 2008). Thus, alterations in the scalp-recorded theta activity are possibly a result of a more complex neuronal network dysfunction. Additionally, both theta and delta activities were shown to reflect EEG slowing due to cholinergic deafferentation of the cortex that is a major neurotransmitter failure in AD and occurs already at the MCI stage of the disease (Spehlmann and Norcross, 1982; Whitehouse et al., 1982; Riekkinen et al., 1990; Lee et al., 1994; Haense et al., 2012). Interestingly, some neuropsychiatric diseases that could also cause memory impairment in a cluster of other clinical features have shown similar alteration in the GFS. For example, in obsessive-compulsive disorder, a decreased GFS in delta, theta, and the slow alpha band was reported (Özçoban et al., 2018). Decreased GFS in theta-frequency band was also reported in first-episode, neuroleptic-naïve patients with schizophrenia (Koenig et al., 2001). In healthy subjects, a simple working memory activation paradigm increases the activity in the theta band (Gevins et al., 1997). Decreased functional synchrony in the theta band in resting-state EEG of cognitively impaired subjects might therefore reflect disease-induced desynchronization of neuronal networks that are necessary for successful performance of the working memory task. In addition, a number of other studies showed that scalp-recorded theta power and synchronization in humans correlated with cognitive processing involved in encoding and retrieving verbal stimuli (Kahana, 2006).

In contrast, neither of the spectral power-related EEG measures played any significant role in discriminating the two amnestic subtypes of MCI. In previous publications, a temporal pattern of changes in EEG power spectra has been repeatedly confirmed on a continuum of AD, including MCI. The temporal dynamics of EEG power alterations during the course of the disease include an early increase in theta and decrease in beta power, followed by a decrease in alpha and an increase in delta power (Coben et al., 1985; Dierks et al., 1991; Prichep et al., 1994). Interestingly, the recent MEG study by López et al. (2014) showed an increase in delta and theta and a decrease in alpha and beta power in patients with md-aMCI compared with those with sd-aMCI; however, it involved relative power measures on topographical clusters of sensors, thus presenting with some key methodological differences. In addition, Moretti et al. (2009) showed a correlation between the increase of the relative theta/gamma power ratio and performance on memory tests in subjects with MCI. Another study that assessed changes in topographical resting-state EEG sources between different MCI subtypes showed increased occipital theta and decreased centro-parieto-occipital alpha activity in amnestic compared with non-amnestic MCI. The same study observed a positive correlation between central-parietal alpha and a negative correlation between frontal delta sources and scores on cognitive tests assessing attention, episodic memory, and executive functions (Babiloni et al., 2010). However, it may not be surprising that global spectral power parameters do not play a role in discriminating the two clinical entities with amnestic profiles and similar low grades of functional impairment since our study did not include cognitively healthy individuals or patients with more severe stages of AD as contrast groups. Rather, our study included patients with MCI at an intermediate cognitive level of impairment, with minimal differences in global cognition (MMSE) between MCI groups, despite showing different cognitive profiles (i.e., single- vs. multiple-domain impairments). This is supported by the low importance of MMSE to discriminate the two MCI groups in our multivariate analysis. In addition, inclusion of the local relative EEG power measures instead of the global parameters that summarize the amplitude/power across all EEG channels may be more sensitive to the fine EEG power changes between MCI subtypes as indicated by some of the previous studies (Moretti et al., 2009; López et al., 2014). Thus, in contrast to GFS that seems to be a trait marker of AD-related early functional disconnection of neuronal networks, differential alterations in global EEG power frequency spectra seem to be a state marker of disease progression.

It is interesting that a novel CSF molecular marker of synaptic pathology, i.e., neurogranin, did not considerately contribute to discriminating the two MCI groups in our multivariate model. This implies that changes in neurophysiological markers of synaptic dysfunction in sd-aMCI, a group that represents prodromal AD, may precede changes in markers of molecular synaptic pathology. Another explanation could be that neurogranin is associated with conditions with memory-related deficits irrespective of AD pathology as was suggested in a study that found that this synaptic marker is also sensitive to age-related cognitive performance on memory tests in neurologically healthy older adults (Casaletto et al., 2017). However, this was contradicted by studies suggesting that the CSF neurogranin is specific to AD-type synaptic dysfunction (Wellington et al., 2016; Portelius et al., 2018).

It is interesting that the biological profile of clinically defined sd-aMCI in this study is closer to the biological profile of AD in contrast to the empirically data-driven classification of sd-aMCI recently published by Edmonds et al. (2021). This discrepancy emphasizes a need for validation of different diagnostic criteria in diverse clinical populations. It is of utmost importance to characterize this prodromal AD stage as early and as accurately as possible to convey the risk and likelihood of developing AD dementia to patients (Frederiksen et al., 2021).

A limitation of our multivariate model is the small groups size, especially for the patients with md-aMCI (*n* = 30) when the cohort is split in 70% for training and 30% for testing of model performance. However, the multivariate model in this study was designed as an extension of the univariate tests for group differences, to investigate EEG measures and CSF biomarkers in the context of age, sex, education, and MMSE measures. Both set of analyses converged in the findings, validating the results from the multivariate model despite the small group size for the test set. Another limitation is that the non-memory cognitive domains affected in the md-aMCI group may vary from patient to patient. Hence, our current results could be expanded in future studies with a larger md-aMCI group, by analyzing associations of different non-memory cognitive domains with qEEG and CSF biomarkers. Importantly, the inclusion of the control group in such comparisons may extend the panel of relevant qEEG and CSF biomarkers for contrasting different cognitive subtypes and considerably add to the interpretation of the results when it comes to the expected direction of change from the cognitively healthy state. Furthermore, the analysis of GFS measure over full EEG frequency spectra instead of averaging across standard frequency bands, or a local topographical parcellation of synchronization patterns, may provide more detailed and physiologically meaningful results in this patient group. Addition of the analysis in the gamma-frequency range while addressing high-frequency artifact contamination would be of further interest since gamma oscillations have been associated with different cognitive processes and were shown to be impaired in AD (Herrmann and Demiralp, 2005; van Deursen et al., 2008; Zheng et al., 2016; Etter et al., 2019).

In conclusion, our study suggests that measures of global EEG synchronization could contribute to the characterization of synaptic dysfunction in different MCI cognitive subtypes. Future studies are required to address and further explore some of the limitations of this study by including other clinical and etiological subtypes of MCI as well as cognitively healthy subjects.

## Data Availability Statement

The original contributions presented in the study are included in the article, further inquiries can be directed to the corresponding author.

## Ethics Statement

This study was approved by the local ethical committee of the Karolinska Hospital and Regional Ethical Review Board in Stockholm (Dnr: 2020-00678, 2011/1978-31/4). Written informed consent for participation was not required for this study in accordance with the national legislation and the institutional requirements.

## Author Contributions

US, DF, and VJ: conceptualization. US, DF, BA, TK, NA, HZ, KB, and VJ: methodology. US, DF, and BA: formal analysis. US, BA, and VJ: investigation. VJ and US: resources, writing—original draft preparation, and funding acquisition. VJ, DF, BA, NA, HZ, and VJ: writing—review and editing. US and DF: visualization. VJ: supervision. All authors have read and agreed to the published version of the manuscript.

## Conflict of Interest

The authors declare that the research was conducted in the absence of any commercial or financial relationships that could be construed as a potential conflict of interest.

## Publisher’s Note

All claims expressed in this article are solely those of the authors and do not necessarily represent those of their affiliated organizations, or those of the publisher, the editors and the reviewers. Any product that may be evaluated in this article, or claim that may be made by its manufacturer, is not guaranteed or endorsed by the publisher.

## Footnotes

1
www.R-project.org
